# Fibroblast Growth Factor 9 Regulation by MicroRNAs Controls Lung Development and Links *DICER1* Loss to the Pathogenesis of Pleuropulmonary Blastoma

**DOI:** 10.1371/journal.pgen.1005242

**Published:** 2015-05-15

**Authors:** Yongjun Yin, Angela M. Castro, Marrit Hoekstra, Thomas J. Yan, Ajay C. Kanakamedala, Louis P. Dehner, D. Ashley Hill, David M. Ornitz

**Affiliations:** 1 Department of Developmental Biology, Washington University School of Medicine, St. Louis, Missouri, United States of America; 2 Department of Pathology, Children’s National Medical Center, Washington, D.C., United States of America; 3 Lauren V. Ackerman Division of Surgical Pathology, Washington University School of Medicine, St. Louis, Missouri, United States of America; University of Wisconsin, UNITED STATES

## Abstract

Pleuropulmonary Blastoma (PPB) is the primary neoplastic manifestation of a pediatric cancer predisposition syndrome that is associated with several diseases including cystic nephroma, Wilms tumor, neuroblastoma, rhabdomyosarcoma, medulloblastoma, and ovarian Sertoli-Leydig cell tumor. The primary pathology of PPB, epithelial cysts with stromal hyperplasia and risk for progression to a complex primitive sarcoma, is associated with familial heterozygosity and lesion-associated epithelial loss-of-heterozygosity of *DICER1*. It has been hypothesized that loss of heterozygosity of *DICER1* in lung epithelium is a non-cell autonomous etiology of PPB and a critical pathway that regulates lung development; however, there are no known direct targets of epithelial microRNAs (miRNAs) in the lung. Fibroblast Growth Factor 9 (FGF9) is expressed in the mesothelium and epithelium during lung development and primarily functions to regulate lung mesenchyme; however, there are no known mechanisms that regulate FGF9 expression during lung development. Using mouse genetics and molecular phenotyping of human PPB tissue, we show that FGF9 is overexpressed in lung epithelium in the initial multicystic stage of Type I PPB and that in mice lacking epithelial *Dicer1*, or induced to overexpress epithelial *Fgf9*, increased *Fgf9* expression results in pulmonary mesenchymal hyperplasia and a multicystic architecture that is histologically and molecularly indistinguishable from Type I PPB. We further show that miR-140 is expressed in lung epithelium, regulates epithelial *Fgf9* expression, and regulates pseudoglandular stages of lung development. These studies identify an essential miRNA-FGF9 pathway for lung development and a non-cell autonomous signaling mechanism that contributes to the mesenchymal hyperplasia that is characteristic of Type I PPB.

## Author Summary

Pleuropulmonary Blastoma (PPB) is a pediatric disease that presents with multifocal cystic lung lesions. Familial mutations in *DICER1*, an essential gene for microRNA synthesis, predisposes to PPB and other related diseases. Loss or mutation of the second copy of *DICER1* in developing airway epithelium is thought to initiate cyst formation and increase growth of the underlying mesenchyme. In later stages, additional genetic events in PPB mesenchyme (mutations in the DICER1 RNase IIIb domain or in TP53) can lead to the formation of high-grade sarcomas. We hypothesized that loss of DICER1 function in lung epithelium leads to persistent overgrowth of mesenchyme (and subsequent risk for malignancy), implicating an indirect tumor initiation mechanism. In this study, we show histological and molecular similarity in Type I PPB and mice lacking epithelial *Dicer1* or overexpressing epithelial Fibroblast Growth Factor 9 (*Fgf9*), demonstrate increased *FGF9* expression in Type I PPB and in *Dicer1*-deficient mouse lung epithelium, and show that *Fgf9* mediates at least some of the pathology resulting from *Dicer1* inactivation in lung epithelium. Finally, we show that specific lung epithelial microRNAs regulate *Fgf9*. These studies identify FGF9 as a target of DICER1 in lung epithelium that functions as an initiating factor for PPB.

## Introduction

Fibroblast Growth Factor 9 (FGF9) is required during lung development for mesenchymal growth and epithelial branching, and inactivation of *Fgf9* in mice results in perinatal death due to respiratory insufficiency [1–3]. Overexpression of FGF9 in embryonic mouse lung epithelium results in cystic expansion of the small airspaces, increased mesenchymal proliferation, and diminished mesenchymal differentiation [2, 3]. Interestingly, the phenotype of lungs that overexpress FGF9 during development closely resemble those seen in mouse lungs lacking epithelial *Dicer1*, an RNase III protein that is required for the biogenesis of microRNAs (miRNAs), small RNA molecules that most commonly bind to specific sequences in the 3’ UTR of mRNA molecules targeting them for destruction or inhibiting their translation [2, 4–6]. The phenotypic similarity between overexpression of *Fgf9* and loss of epithelial *Dicer1* suggested that microRNA modulation of *Fgf9* expression could be an essential mechanism regulating lung development and that deregulation of *Fgf9* expression could lead to developmental abnormalities or other diseases.

Pleuropulmonary blastoma (PPB), the most common primary malignancy of the lung in children, is either solitary or multifocal, is often familial, and is seen in association with several nonpulmonary neoplasms including cystic nephroma, Wilms tumor, neuroblastoma, rhabdomyosarcoma, medulloblastoma, ovarian Sertoli-Leydig cell tumor, intestinal polyps, and thyroid nodules [7–15]. The earliest morphologic changes in the affected lung consist of a localized area(s) of cystic expansion of alveolar spaces and uncommitted small mesenchymal cells within the expanded alveolar septa (Type I PPB). The interstitial mesenchyme in these early lesions resembles that of the late pseudoglandular stage of lung development [16]. The initial multicystic lung lesion in Type I PPB can progress to a complex primitive sarcoma, which is first recognized by the presence of primitive mesenchymal cells, often with rhabdomyosarcomatous features, arranged in a dense layer (cambium) beneath a benign appearing epithelium. The risk for progression in a purely cystic Type I PPB peaks in the first 5 years of life [16, 17].

Genetic linkage analysis of familial PPB and related disorders identified loss-of-function mutations in *DICER1* [8, 18]. DICER1 is normally expressed in both epithelial and mesenchymal tissues of human (and mouse) lung [18, 19]. Immunostaining for DICER1 showed loss of, or decreased, staining in PPB-associated lung epithelium in a proportion of Type I PPBs but consistent expression in the underlying mesenchyme [18]. We hypothesized that the epithelial and mesenchymal phenotype of early PPB results from focal loss of functional DICER1 in lung epithelium and that deregulation of an epithelial factor would non cell-autonomously affect subepithelial mesenchyme. Of several secreted signaling molecules that are important for lung development, we considered FGF9 a plausible candidate as it is expressed in lung epithelium and mesothelium in early embryonic development and has the capacity to signal to both mesenchyme, where it regulates proliferation and inhibits differentiation, and epithelium where it affects branching and directly induces epithelial dilation [1–3, 20–22].

Here, we show that the lung phenotype caused by loss of epithelial *Dicer1* is dependent on *Fgf9*, as it can be partially rescued by reducing the gene copy number of *Fgf9*. We also show that the *Fgf9* 3’ UTR is responsive to conserved miRNA-140, miRNA-328, and miR-182, and that miRNA-140 (and miR-328) is an important regulator of lung development. Strikingly, we find that FGF9 is highly expressed in the epithelium of Type I PPBs in humans and in mouse embryonic lung epithelium that conditionally lack *Dicer1*. These studies thus identify FGF9 as a developmentally essential downstream target of epithelial DICER1-cleaved miRNAs during lung development and as a candidate “tumor promoting factor” for PPB.

## Results

### Epithelial Dicer1 inactivation results in FGF9-dependent mesenchymal hyperplasia

**Fig 1:**
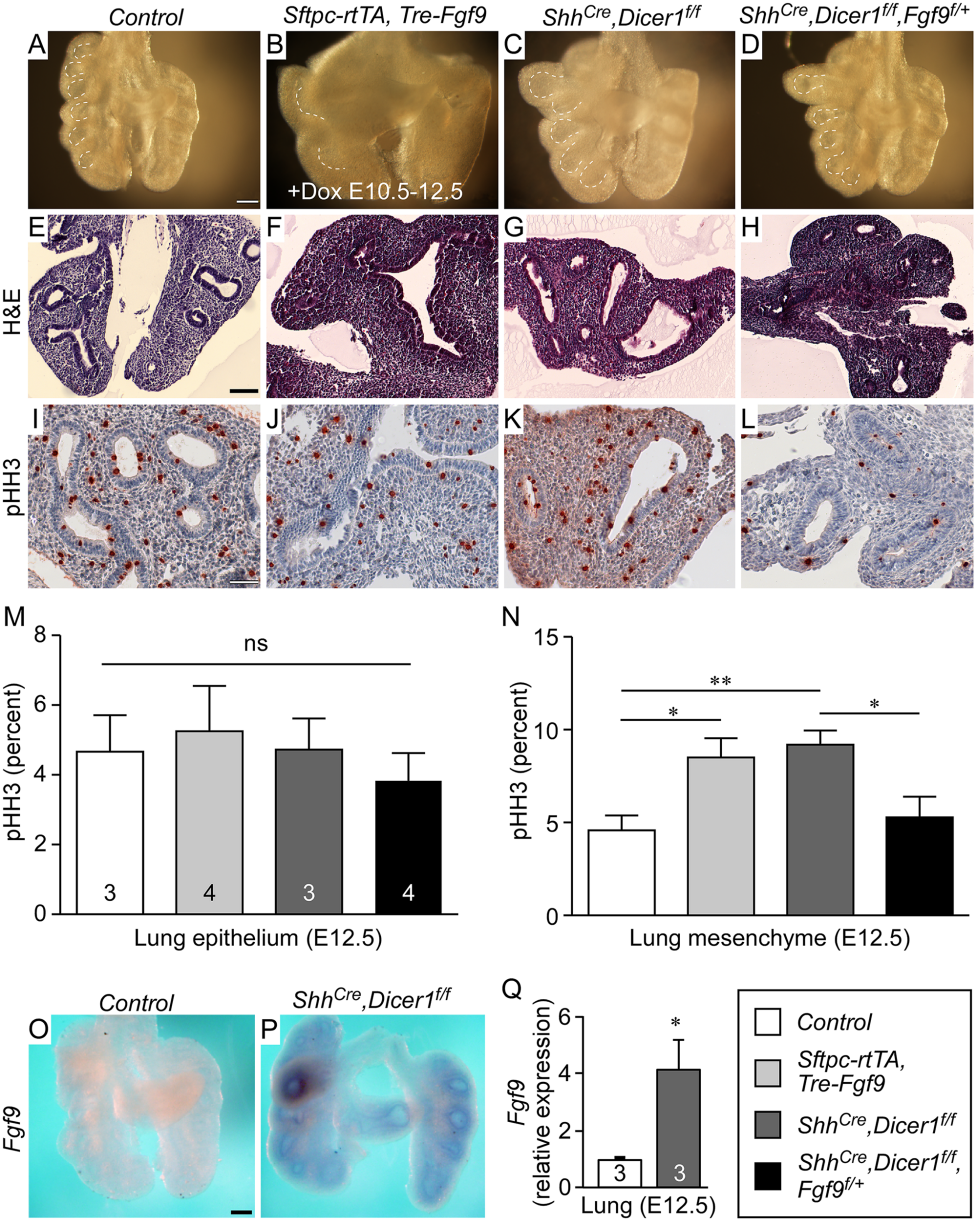
Dicer1 regulation of lung epithelial development requires *Fgf9*. (A-D) Comparison of E12.5 whole mount lung morphology of *Control* (A), *Sftpc-rtTA*, *Tre-Fgf9-Ires-eGfp* lungs induced with doxycycline from E10.5-E12.5 (B), *Shh* ^*Cre/+*^, *Dicer1* ^*f/f*^ (C) and *Shh* ^*Cre/+*^, *Dicer1* ^*f/f*^, *Fgf9* ^*f/+*^ (D). (E-H) H&E stained histological sections of the lungs shown above in panels A-D. (I-L) Representative immunostaining for phospho-histone H3 (pHH3) of the lungs shown in panels A-D. (M and N) Quantification of epithelial (M) and mesenchymal (N) cell proliferation of pHH3 labeled lung tissue in *Control*; *Sftpc-rtTA*, *Tre-Fgf9-Ires-eGfp* lungs induced with doxycycline from E10.5-E12.5; *Shh* ^*Cre/+*^, *Dicer1* ^*f/f*^ lungs; and *Shh* ^*Cre/+*^, *Dicer1* ^*f/f*^, *Fgf9* ^*f/+*^ lungs. For each group, at least 3 individual samples were included, 3 different slides were chosen from each sample, and for each section, three 10x fields were counted for the number of positive cells per 100 cells. (O and P) Whole mount *in situ* hybridization showing increased expression of *Fgf9* in E12.5 *Shh* ^*Cre/+*^, *Dicer1* ^*f/f*^ lung epithelium (P) compared to control lung (O). (Q) Quantitative RT-PCR showing increased expression of *Fgf9* in E12.5 *Shh* ^*Cre/+*^, *Dicer1* ^*f/f*^ lung (n = 3) epithelium compared to control lung (n = 3). *P<0.05; **P<0.01; ns, not significant. Scale bars: A, 200 μm; E, 100 μm; I, 50 μm; O, 200 μm. Sample numbers (n) are indicated in data bars.

To determine whether lung epithelial microRNAs regulate molecules that affect lung mesenchyme development, we used a *Shh* ^*Cre*^ knockin allele [23] to inactivate a floxed allele of *Dicer1* [24] specifically in lung epithelium. At embryonic day 10.5 (E10.5), *Shh* ^*Cre/+*^, *Dicer1* ^*f/f*^ lungs were histologically and phenotypically normal; however, E12.5, *Shh* ^*Cre/+Cre/+/+*^, *Dicer1* ^*f/f*^ lungs were larger than controls, with dilated epithelial ducts, reduced branching, and substantially expanded mesenchyme (Fig 1A, 1C, 1E, and 1G). E14.5 and E16.5, *Shh* ^*Cre/+*^, *Dicer1* ^*f/f*^ lungs were of similar size to controls but revealed marked cystic expansion of the epithelial ducts and decreased branching (S1 Fig). Because of the early inactivation of *Dicer1* with *Shh* ^*Cre/+*^ and the rapid progression of the phenotype, we focused most subsequent analyses on the E12.5 time point.

The striking expansion of mesenchyme in lungs lacking epithelial *Dicer1* suggested activation of a non-cell autonomous epithelial-derived signal. Because of the established role of FGF9 signaling to lung mesenchyme [1, 2], we considered *Fgf9* as a plausible candidate for this signal. *In situ* hybridization and qRT-PCR examination of E12.5 lung showed increased *Fgf9* expression, primarily localized to epithelium, in *Shh* ^*Cre/+*^, *Dicer1* ^*f/f*^ compared to control lungs (Fig 1O–1Q). To compare phenotypes resulting from ectopic overexpression of epithelial *Fgf9* with epithelial loss of *Dicer1*, we forced expression of FGF9 in lung epithelium by crossing the *Sftpc-rtTA* transgenic mouse line with mice containing a doxycycline inducible *Fgf9* transgene, *Tre-Fgf9-Ires-Gfp* (*Tre-Fgf9*). These double transgenic mice could be induced to express FGF9 in lung epithelium in the presence of doxycycline [2]. Induction of FGF9 expression from E10.5 to E12.5 showed a similar phenotype to lungs lacking epithelial *Dicer1* (Fig 1B, 1C, 1F, and 1G). Consistent with induction of a primary epithelial to mesenchymal signal in both *Shh* ^*Cre/+*^, *Dicer1* ^*f/f*^ lungs and doxycycline-induced *Sftpc-rtTA*, *Tre-Fgf9-Ires-eGfp* lungs, analysis of cell proliferation showed no significant change in epithelial proliferation at E12.5, but significantly increased mesenchymal proliferation (Fig 1I–1K, 1M, and 1N).

**Fig 2:**
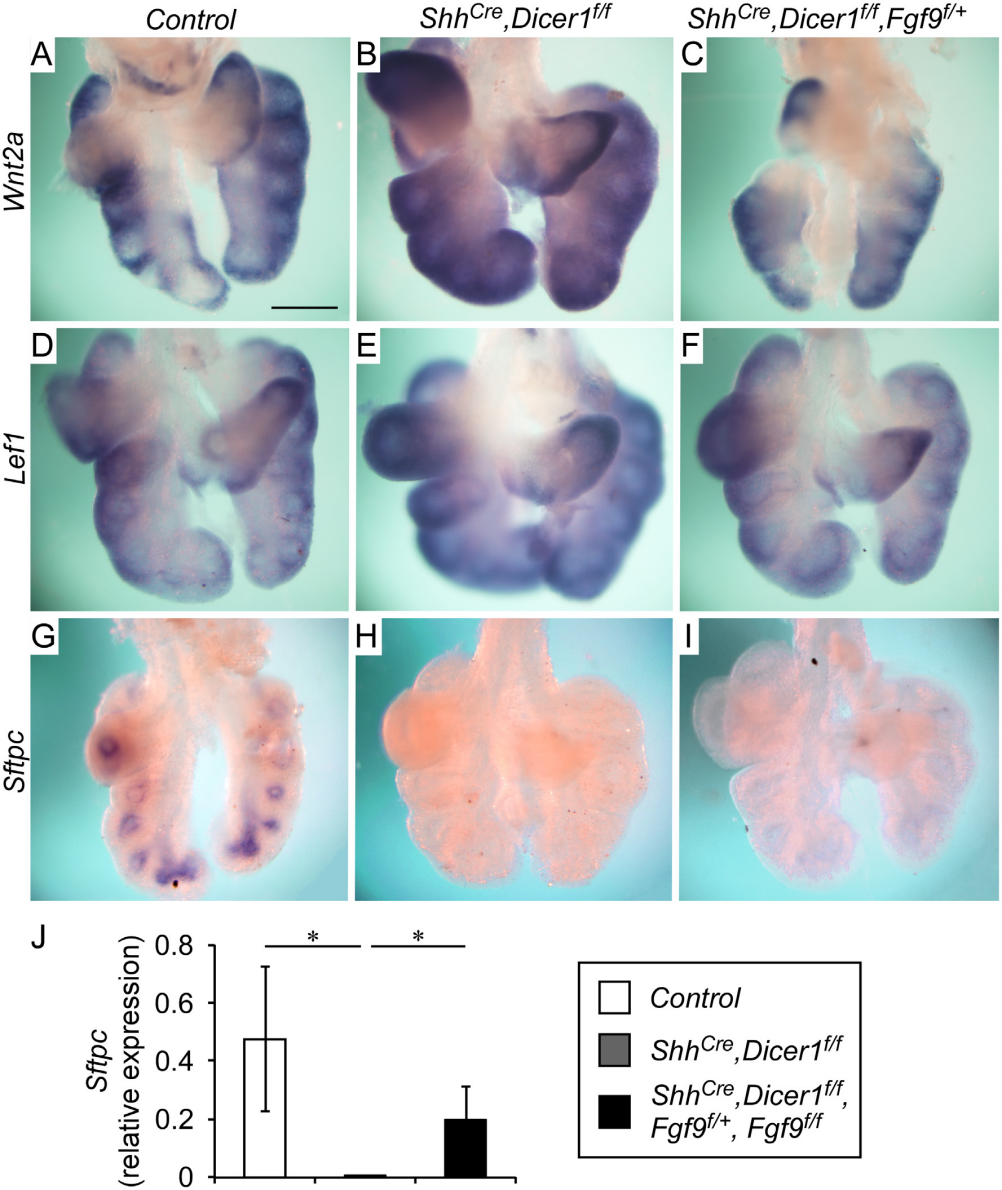
Epithelial Dicer1 regulation of mesenchymal Wnt/β-Catenin signaling and epithelial differentiation requires *Fgf9*. (A-C) Expression of *Wnt2a* in E12.5 lung showing increased expression in *Shh* ^*Cre/+*^, *Dicer1* ^*f/f*^ distal lung mesenchyme (B) compared to the Control (A). Inactivation of one allele of *Fgf9* in *Shh* ^*Cre/+*^, *Dicer1* ^*f/f*^ lung epithelium (C) reduces *Wnt2a* expression to levels observed in control lungs. (D-F) The downstream target of Wnt signaling, *Lef1*, was increased in *Shh* ^*Cre/+*^, *Dicer1* ^*f/f*^ lung mesenchyme (E) compared to the Control (D). Inactivation of one allele of *Fgf9* in *Shh* ^*Cre/+*^, *Dicer1* ^*f/f*^ lung epithelium (F) reduces *Lef1* expression to levels observed in control lungs. (G-I) Expression of *Sftpc* in E12.5 lung showing decreased expression in *Shh* ^*Cre/+*^, *Dicer1* ^*f/f*^ distal lung mesenchyme (H) compared to the Control (G). Inactivation of one allele of *Fgf9* in *Shh* ^*Cre/+*^, *Dicer1* ^*f/f*^ lung epithelium (I) results in increased *Sftpc* expression. (J) Quantitative PCR analysis of E12.5 Control lung and *Shh* ^*Cre/+*^, *Dicer1* ^*f/f*^ rescued with one or two Fgf9 floxed alleles showing increased Sftpc expression. * *P*<0.04. Images shown are representative of three embryos for each genotype. Scale bars: 200 μm.

Previous studies on lung development identified a feed forward signaling network that linked FGF9 with mesenchymal FGFR and Wnt/β-Catenin signaling and *Fgfr1* and *Fgfr2* expression [3, 25]. In this network, FGF9 and mesenchymal FGFR signaling regulated the expression of the canonical Wnt ligand, *Wnt2a*, and activated mesenchymal Wnt/β-Catenin signaling. Mesenchymal Wnt/β-Catenin signaling, driven by mesenchymal *Wnt2a* and epithelial *Wnt7b*, was required for the expression of mesenchymal *Fgfr1* and *Fgfr2* [3, 21]. To determine if this feed forward loop was activated in lung tissue lacking epithelial *Dicer1*, we examined expression of *Wnt2a* and the WNT-responsive transcription factor, *Lef1*. Compared to control lung (Fig 2A and 2D), *Shh* ^*Cre/+*^, *Dicer1* ^*f/f*^ lungs showed increased *Wnt2a* and *Lef1* expression (Fig 2B and 2E). These observations support a model in which upregulation of *Fgf9* in *Shh* ^*Cre/+*^, *Dicer1* ^*f/f*^ lungs activate mesenchymal FGF-Wnt/β-Catenin signaling.

### Rescue of epithelial *Dicer1* deficiency by reduction in epithelial *Fgf9* gene dosage

If increased *Fgf9* expression were a primary factor mediating the phenotype resulting from epithelial inactivation of *Dicer1*, partial rescue would be expected following epithelial-specific reduction of *Fgf9* gene dosage. To test this, we generated mouse embryos with the genotype *Shh* ^*Cre/+*^, *Dicer1* ^*f/f*^, *Fgf9* ^*f/+*^ in which one allele of *Fgf9* was inactivated specifically in lung epithelium that also lacked both copies of *Dicer1*. By itself, heterozygosity for *Fgf9* has no effect on development [1–3]. Compared to *Shh* ^*Cre/+*^, *Dicer1* ^*f/f*^ littermates, genetic inactivation of one allele of *Fgf9* in lung epithelium significantly reduced lung size and epithelial dilation (Fig 1C and 1D). Examination of lung histology revealed reduction in both epithelial airspace dilation and mesenchymal thickness (Fig 1G and 1H). Immunostaining for phospho-Histone H3 (pHH3) positive cells showed a reduction in mesenchymal proliferation in *Shh* ^*Cre/+*^, *Dicer1* ^*f/f*^, *Fgf9* ^*f/+*^ compared to *Shh* ^*Cre/+*^, *Dicer1* ^*f/f*^, *Fgf9* ^*+/+*^ lungs (Fig 1K, 1L and 1N). Consistent with the normalized phenotype resulting from inactivation of one allele of epithelial *Fgf9*, *Wnt2a* and *Lef1* expression levels were also normalized in *Shh* ^*Cre/+*^, *Dicer1* ^*f/f*^, *Fgf9* ^*f/+*^ lung tissue (Fig 2A–2F). We also examined potential phenotypic rescue of lung tissue lacking both alleles of *Fgf9* in epithelium of mice lacking epithelial *Dicer1* (*Shh* ^*Cre/+*^, *Dicer1* ^*f/f*^, *Fgf9* ^*f/f*^). At E14.5, epithelial inactivation of both alleles of *Fgf9* (in the *Shh* ^*Cre/+*^, *Dicer1* ^*f/f*^ background) resulted in a smaller lung with reduced cystic dilation of epithelial ducts (S2 Fig). This finding is consistent with inactivation of epithelial FGF9 compensating for the epithelial *Dicer1* loss phenotype and endogenous mesothelial *Fgf9* (which is not affected in this model) having a primary role in regulating lung mesenchymal development [21].

To further assess the contribution of FGF9 signaling to early stages of epithelial differentiation, we examined expression of *Sftpc*, an epithelial differentiation marker, which is first expressed in the epithelial branching tips at ~E12.5. Compared to control lung, *Shh* ^*Cre/+*^, *Dicer1* ^*f/f*^ lungs showed significantly reduced expression of *Sftpc* at E12.5 (Fig 2G and 2H). However, in *Shh* ^*Cre/+*^, *Dicer1* ^*f/f*^, *Fgf9* ^*f/+*^ lung, low-level expression of *Sftpc* was detected (Fig 2I) consistent with partial rescue of lung development. Quantitative RT-PCR on whole E12.5 lung also demonstrated a significant (*P*<0.04) increase in the expression of *Sftpc* in *Shh* ^*Cre/+*^, *Dicer1* ^*f/f*^, *Fgf9* ^*f/+*^ *(or Fgf9* ^*f/f*^ *)* lung compared to control lung (Fig 2J).

### *FGF9* is regulated by miRNAs that are expressed in developing lung epithelium

**Fig 3:**
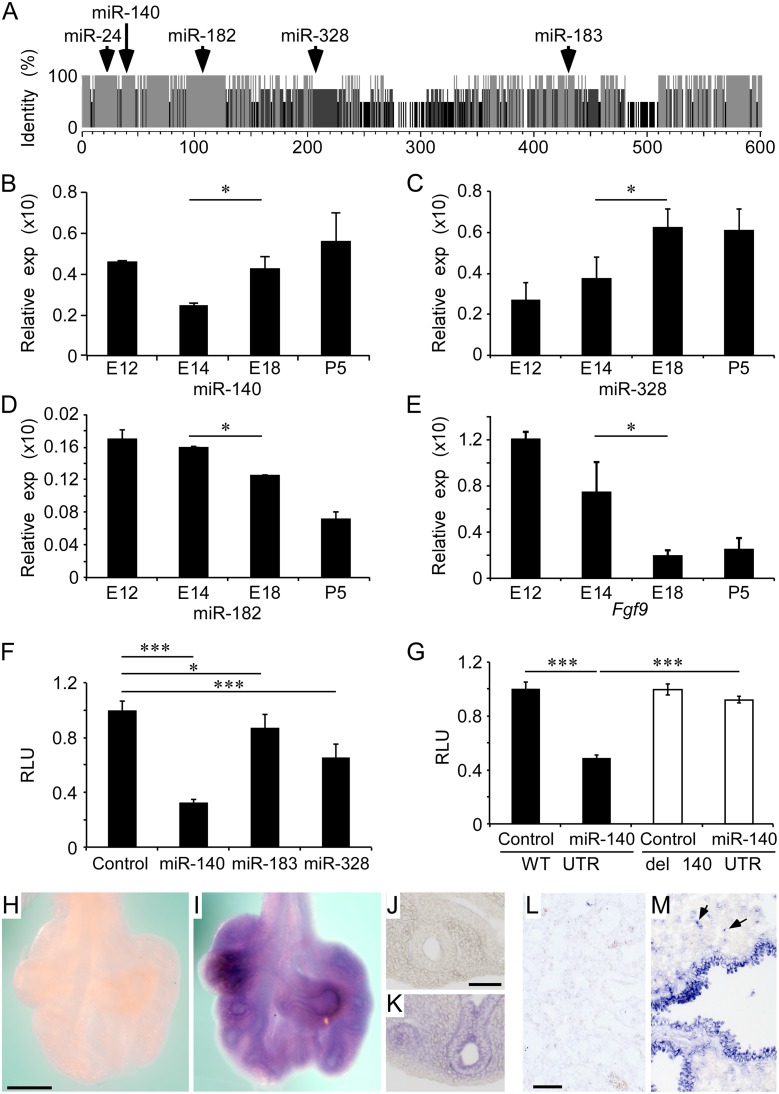
Regulation of the *Fgf9* 3' UTR by miRNAs expressed in developing lung epithelium. (A) Conservation of miRNA binding sites by comparison of *Fgf9* 3’ UTR sequences from human, chimp, mouse, and pig. Grey, 100%; Dark grey, 75%; Black, 50% sequence identity. The position of specific miRs that are also expressed and regulated during lung development are indicated. (B-D) Relative developmental expression profile at E12.5, E14.5, E18 and P5 (normalized to *U6 snRNA* or *Hprt*) of miR-140 (B), miR-328 (C), miR-182 (D), and *Fgf9* (E). (F) Repression of luciferase activity by miR-140, miR-183, and miR-328 double stranded miRNA mimics compared to a cel-miR-67 control double stranded miRNA mimic (S3 Fig, S2 Table), when co-transfected in HEK293 cells with a luciferase reporter construct containing a wild type mouse *Fgf9* 3’ UTR. (G) Repression of the *Fgf9* 3’ UTR by miR-140 (solid bars) was blocked by mutations that deleted the seed sequences for miR-140 (open bars). (H-M) *In situ* hybridization to localize expression of miR-140 in E12.5 and E18.5 lung. E12.5 lung was hybridized with a scrambled LNA *in situ* probe (H) or with the hsa-miR-140-5p LNA probe (I) (S2 Table). J and K are frozen sections from the tissue in H and I, respectively. Histological sections from E18.5 wild type mouse lung were hybridized with a scrambled LNA *in situ* probe (L) or with hsa-miR-140-5p LNA probe (M). Arrows in (M) indicate patterns that are consistent with expression in type II pneumocytes. All data is derived from at least 3 independent experiments. *P<0.05, *** *P*<0.001. Scale bars: H, 200 μm; J and L, 50 μm.

Sequence analysis of the *FGF9* 3’ UTR identified multiple potential miRNA binding sites that were highly conserved between human, chimp, mouse, and pig (Fig 3A and S3 Fig). Importantly, several of these miRNAs (miR-24, miR-140, miR-182, miR-183, miR-328) are expressed in fetal or neonatal lung and their relative expression levels are modulated during lung development [26, 27] or in lung cancer [28–30]. Analysis of expression of these miRs at different stages of lung development showed that miR-140-5p and miR-328-3p were expressed at relatively low levels during the pseudoglandular stage of lung development and at relatively higher levels during the saccular stage, while miR-182-5p showed the opposite profile (Fig 3B–3D). By comparison, *Fgf9* expression levels were relatively high during pseudoglandular stage lung development and lower at late developmental stages (Fig 3E).

To functionally assay the *FGF9* 3’ UTR, we cloned the mouse and human UTR’s into the dual luciferase vectors psiCHECK-2 (*pFgf9UTR*) and pEZX-MT01 (*pFGF9UTR*), respectively, and transfected into HEK293 cells. Mature microRNA mimics for miR-24, miR-140, miR-182, miR-183, and miR-328 were then screened for their ability to regulate luciferase activity of the human or mouse *FGF9* 3’ UTR. Of these, miR-140, miR-183, and miR-328 suppressed luciferase activity, while miR24 and miR-182 increased luciferase activity (Fig 3F, mouse, and S4A Fig and S4B Fig, human). Because miR-140 demonstrated the strongest repressive effect on luciferase activity, this miRNA was investigated further.

To establish specificity of miR-140, we engineered mutations in the mouse and human *FGF9* 3’ UTR miR-140 seed sequences. Luciferase activity assays showed that the miR-140 mutant 3’ UTRs no longer responded to co-transfection with the respective mature miR mimic (Fig 3G and S4C Fig). To determine the primary cell-type expressing miR-140, we hybridized locked nucleic acid (LNA) *in situ* probes to E12.5 whole lungs and E18.5 lung sections. Consistent with regulation of epithelial *Fgf9* mRNA expression, miR-140 was prominently expressed in E12.5 lung epithelial ducts and E18.5 distal conducting airway epithelium and Type II pneumocytes (Fig 3H–3M). miR-328 was also prominently expressed in E18.5 lung epithelium (S5 Fig). Collectively, expression patterns and *in vitro* suppression of the *Fgf9* 3’ UTR identified miR-140 and miR-328 as candidate miRNAs that could function *in vivo* to suppress *Fgf9* as lung development progresses from pseudoglandular to canalicular stages.

**Fig 4:**
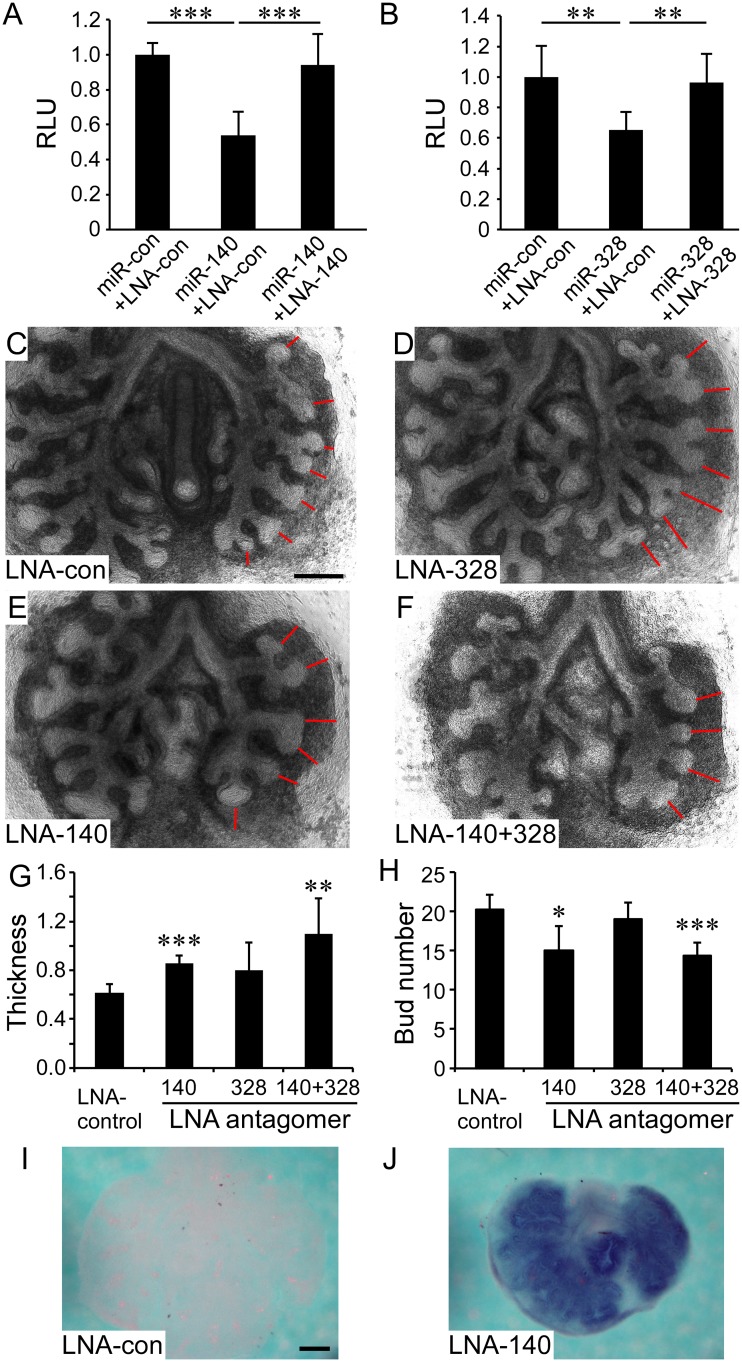
MiR-140 and miR-328 regulate *in vitro* lung development and *Fgf9* expression. (A-B) Validation of tiny LNA antagomers ability to block the activity of miR-140 and miR-328. Repression of the *Fgf9* 3’ UTR by 10 nM miR-140 (A) or 10 nM miR-328 (B) transfected into HEK293 cells with a luciferase reporter construct containing a wild type mouse *Fgf9* 3’ UTR was blocked by adding 10 nM of the corresponding tiny LNAs to the culture medium. The control LNA (LNA-con) contains a single mismatch in the LNA-140 sequence. (C-F) E12.5 lung explants were cultured in the presence of 100 nM tiny LNA oligonucleotides (S2 Table) for 48 hr. (C) Control LNA (100 nM), (D) LNA-328, E) LNA-140, and (F) 50 nM of LNA-140 and LNA-328 (total concentration 100 nM). Red lines indicate mesenchymal thickness. (G and H) Quantification of mesenchymal thickness (G) and the epithelial bud number (H) of lung explants in response to treatment with tiny LNA antagomers (*n* = 4–5 explants per condition). (I and J) Whole mount *in situ* hybridization showing expression of *Fgf9* in E12.5 wild type lung explants cultured in the presence of 100 nM control LNA (I) or LNA-140 (J). Images shown are representative of at least three independent experiments. *P<0.05, **P<0.01, *** *P*<0.001. Scale bars: 200 μm.

To establish whether miR-140 and miR-328 functionally regulate lung development, lung explant cultures were treated with seed-targeting 8-mer LNA oligonucleotides or single mismatch control LNA oligonucleotides (tiny LNAs) [31]. To demonstrate efficacy of tiny LNAs, HEK293 cells, transfected with *pFgf9 UTR* and miR-140 or miR-328 mimics, were co-transfected with tiny LNA antagomers. At a concentration of 10 nM, tiny LNAs effectively blocked miR-140 or miR-328 ability to suppress *Fgf9* 3’ UTR activity *in vitro* (Fig 4A and 4B). Using 6-FAM-labeled miR-140 tiny LNA, we also demonstrated efficient uptake into lung explant tissue 48 hr following exposure to media containing 100 nM tiny LNA (S6 Fig), consistent with efficient uptake of other types of oligonucleotides into lung explant cultures [21].

Embryonic lungs, explanted at E10.5, showed robust mesenchymal growth and epithelial branching over 48 hr in culture. In response to treatment with FGF9, lung explants revealed increased mesenchymal thickness and epithelial airspace dilation [3, 21, 32]. Similar to lung explants treated with FGF9, treatment of E10.5 explants with 100 nM anti-miR-140 or 50 nM of each, anti-miR-140 and anti-miR-328, showed a significant (*P*<0.01) increase in mesenchymal thickness and a decrease in epithelial branching compared to explants treated with mismatch control LNA oligonucleotides (Fig 4C–4H).

Finally, *Fgf9* expression was evaluated in lung explant cultures treated with tiny LNAs. Lungs treated with the LNA-140 (*n* = 4 of 4) or LNA-140 and LNA-328 (n = 4 of 4) demonstrated increased expression of *Fgf9* compared to treatment with control LNA in which only one of seven explants showed *Fgf9* expression (Fig 4I and 4J). These data indicated that during *ex vivo* lung development, miR-140 is sufficient to regulate *Fgf9* expression in lung epithelium and regulate mesenchymal growth and epithelial branching.

### FGF9 is a candidate non-cell autonomous factor in human Type I PPB

**Fig 5:**
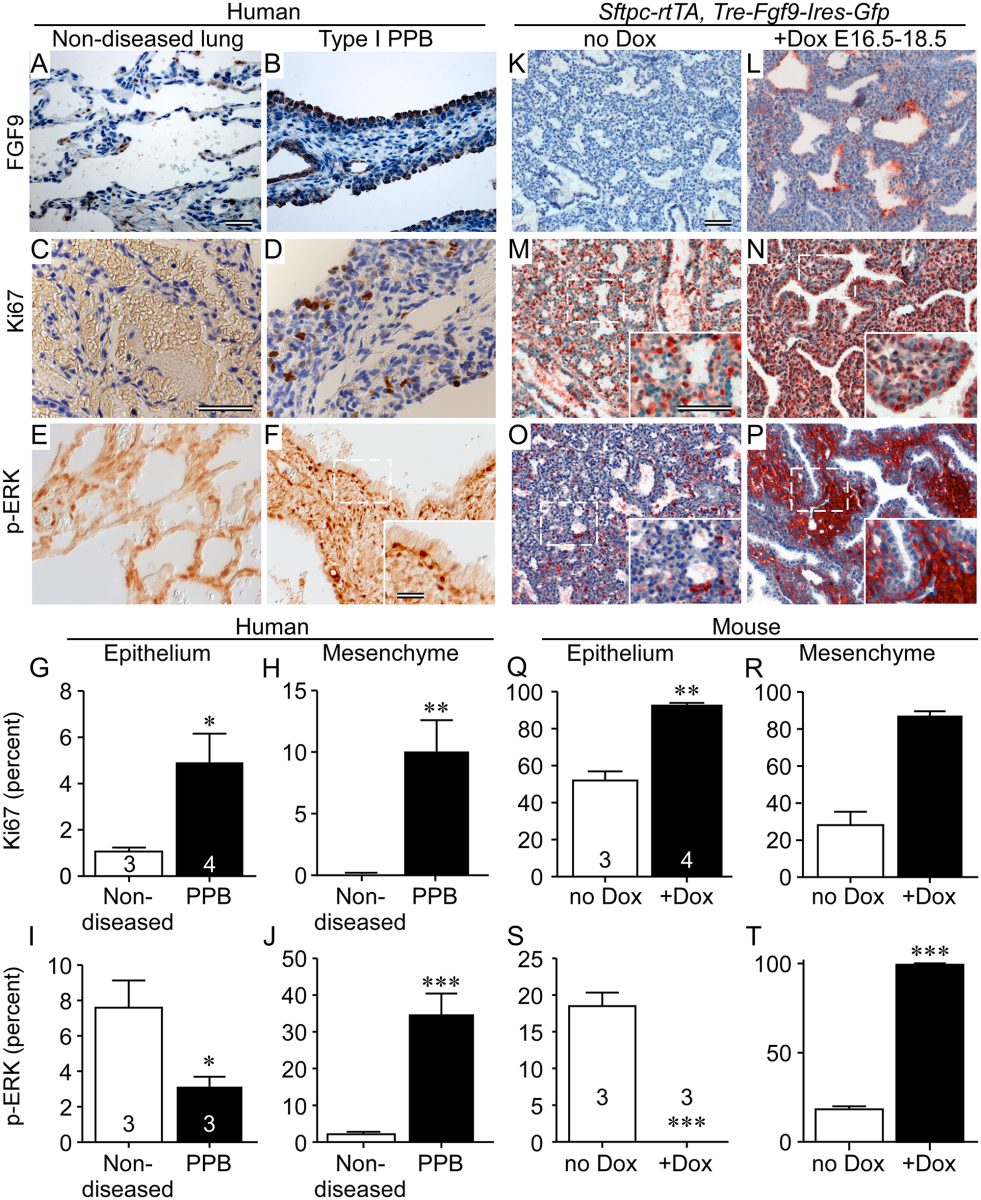
FGF9 overexpression in Type I PPB phenocopies ectopically expressed FGF9 in mouse lung epithelium. (A, B, K, and L) Immunostaining for FGF9 showing increased expression in Type I PPB-associated lung epithelium and in doxycycline-induced *Sftpc-rtTA*, *Tre-Fgf9-Ires-Gfp* mouse lung. Non-diseased human lung and uninduced (no Dox) mouse lung were used as controls. (C, D, M, and N) Mesenchymal and epithelial proliferation in Type I PPB and induced mouse lung identified by immunostaining for Ki67. Inserts show higher magnification of the boxed regions. (E, F, O, and P) Immunostaining showing increased phosphorylated Erk1/2 (p-ERK) in Type I PPB and in induced mouse lung mesenchyme and reduced p-ERK in epithelium. Inserts show higher magnification of the boxed regions. (G, H, Q and R) Quantification of Ki67 immunostaining in C, D, M, and N above, showing increased in proliferation in both epithelial and mesenchymal tissues of Type I PPB (G and H) and *Fgf9*-induced mouse lung (Q and R). (I, J, S, and T) Quantification of p-ERK immunostaining in E, F, O and P above, showing decreased epithelial p-ERK (I and S) and increased mesenchymal p-ERK (J and T), in Type I PPB and *Fgf9*-induced mouse lung compared to control tissue. Error bars represent SD. * *p*<0.05, ** *p*<0.01, *** *p*<0.001. (A and B) Five month-old male; (C and D) Three month-old female; (E) Six month-old female; (F) 34 month-old female. Scale bars: A and B, 20 μm; C-P, 50 μm. Sample numbers (n) are indicated on the data bars.

Identification of *DICER1* loss in Type1 PPB-associated lung epithelium suggests that deregulation of a non-cell autonomous factor could initiate the pathological process leading to abnormal mesenchymal proliferation and subsequent oncogenic transformation. To test this, human Type I PPB tissue was immunostained for FGF9, the proliferation marker, Ki67 and p-Erk. Robust epithelial FGF9 expression was observed in 13 of 16 cases (81%) of Type I PPBs examined (Fig 5A and 5B). Consistent with FGF9 signaling to mesenchyme, cell proliferation, as determined by Ki67 immunostaining and p-ERK expression was increased in subepithelial mesenchyme in all (n = 10) cases assessed (Fig 5C–5F, 5H and 5J). Examination of PPB-associated epithelium showed increased proliferation, but reduced p-ERK immunostaining (Fig 5G and 5I) in nine of eleven (81%) cases assessed, suggesting direct consequences of *DICER1* loss in epithelium and possible indirect effects of FGF9 secondary to increased mesenchymal proliferation. For comparison with similarly staged mouse lung, epithelial FGF9 expression was induced from E16.5 to E18.5 in *Sftpc-rtTA*, *Tre-Fgf9-Ires-Gfp* double transgenic embryos (Fig 5K and 5L). Immunostaining for Ki67 showed increased proliferation compared to uninduced control mouse lung, within mesenchymal and epithelial compartments (Fig 5M, 5N, 5Q and 5R). Similar to what was observed in the tissues of human PPBs, p-ERK expression was significantly increased in lung mesenchyme, but absent in associated epithelium (Fig 5O, 5P, 5S and 5T).

### Histologic and molecular phenotyping show similarities in FGF9 overexpressing mouse lung and human Type I PPB

**Fig 6:**
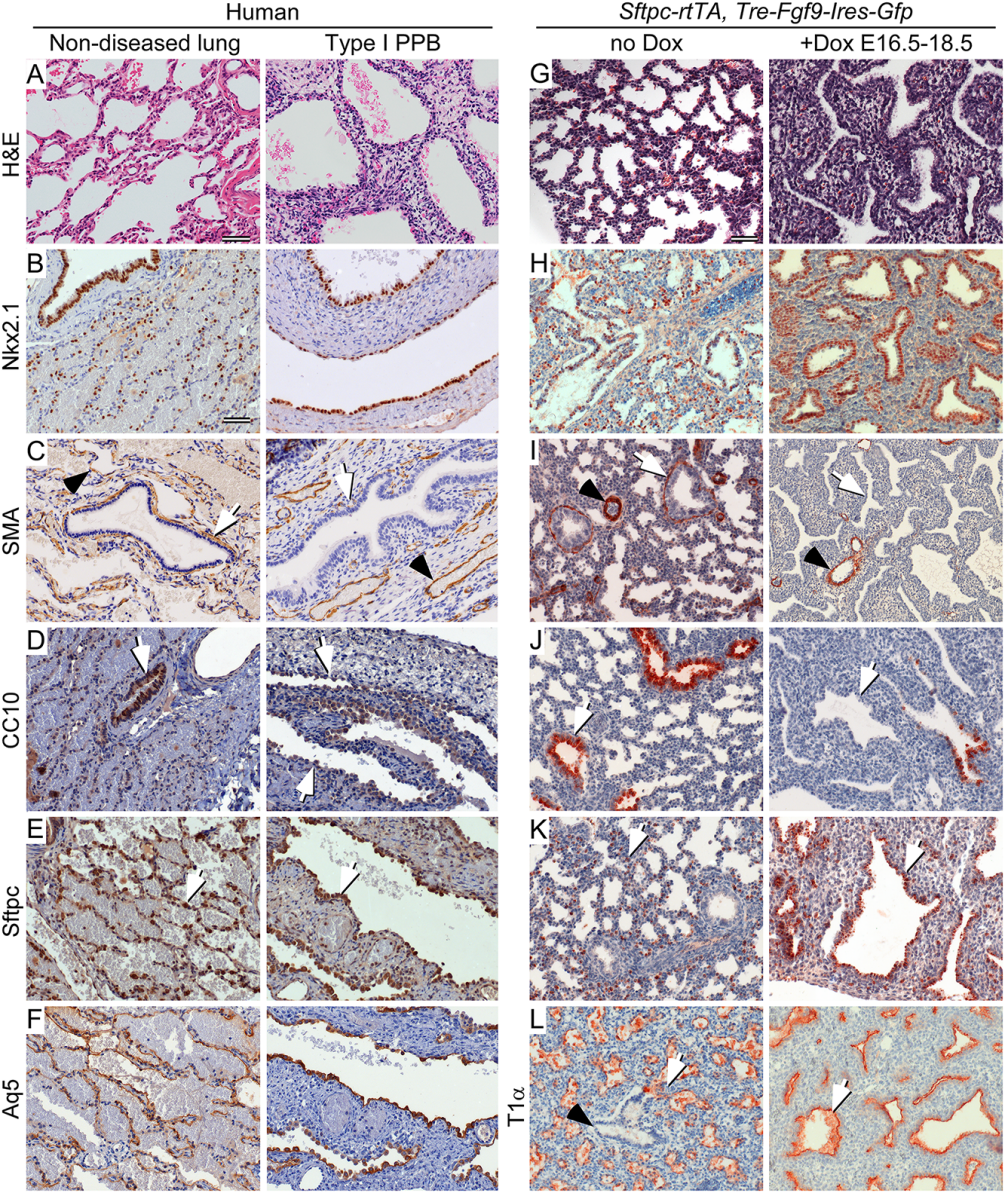
Type I PPB and induced late gestation expression of epithelial FGF9 in mice have similar histopathology and cell differentiation. (A-F) Comparison of non-diseased human lung (left) and Type I PPB (right). (G-L) Comparison of normal (no Dox) E18.5 mouse lung (left) and mouse lung from *Sftpc-rtTA*, *Tre-Fgf9-Ires-eGfp* double transgenic mice induced (+Dox) to overexpress *Fgf9* from E16.5 to E18.5 (right). (A and G) H&E stained histological sections. (B and H) Immunostaining for Nkx2.1 to identify lung epithelium. (C and I) Immunostaining for smooth muscle actin (SMA). Peri-bronchiolar SMA immunostaining (white arrow) and perivascular SMA immunoreactivity (black arrowhead) are differentially affected. (D and J) Immunostaining for Club cell secretory protein (CC10) showing reduced expression of CC10 in proximal lung epithelium (white arrow) compared to that of non-diseased human lung and uninduced mouse lung, respectively. (E and K) Immunostaining for surfactant protein C (Sftpc) showing expanded proximal expression (white arrow) in all cystic lung epithelium in human Type I PPB and *Fgf9*-induced mouse lung. In non-diseased human lung and uninduced mouse lung, Sftpc immunostaining (white arrow) was consistent with expression in Type II pneumocytes. (F and L) Immunostaining for the distal lung Type I pneumocyte marker Aquaporin 5 (Aq5, human) and T1α (mouse). In human, Aq5 was expressed in distal alveoli of non-diseased lung tissue and in Type I PPB associated epithelium. T1α was similarly expressed in distal mouse lung and throughout the epithelium of *Fgf9*-induced mouse lung (white arrow). (A) Three month-old female. (B-F) Three month-old female. Scale bar: A, 20 μm, B-L, 50 μm.

The pathologic findings in Type I PPB showed a characteristic expansion of primitive or uncommitted mesenchyme (Fig 6A) associated with a benign-appearing Nkx2.1 positive epithelium (Fig 6B). In the mouse, induction of epithelial FGF9 expression from E16.5 to E18.5 resulted in mesenchymal hyperplasia beneath a benign-appearing Nkx2.1 positive epithelium (Fig 6G and 6H) with histological features that were virtually identical to those observed in Type I or cystic PPB (Fig 6A and 6B). Control double transgenic embryos that were not exposed to doxycycline were phenotypically normal. Both in human Type I PPB and in mouse lung induced to express FGF9, the continued expression of Nkx2.1 indicates that lung epithelial identity is retained.

Progression towards malignancy often involves loss of cellular terminal differentiation. Examination of mesenchymal differentiation into peribronchiolar smooth muscle showed reduced expression of smooth muscle actin (SMA) in peribronchiolar locations in both Type I PPB tissue and mouse lung tissue induced to express FGF9 (Fig 6C and 6I, white arrow). However, vascular SMA expression appeared normal in both mouse and human tissue (Fig 6I, black arrowhead). Markers of proximal-distal epithelial differentiation were similarly altered in both human Type I PPB and FGF9-induced mouse lung. The proximal Club cell secretory protein, CC10, expression was decreased in bronchiolar epithelium (Fig 6D and 6J, white arrow), the distal alveolar Type II cell marker, Sftpc (Fig 6E and 6K, white arrow) and the alveolar Type I cell marker Aquaporin 5 (Aq5) [33] or T1α [34] (Fig 6F and 6L, white arrow) were increased and expanded proximally. Thus, loss of *DICER1* in human lung epithelial tissue or overexpression of FGF9 in late stage fetal mouse lung epithelium not only affects mesenchymal growth and differentiation, but also results in distal differentiation of epithelial cell types. The decreased expression of Sftpc in E12.5 lungs that lack Dicer1 (Fig 2G,2H and 2J) likely represents a delay in epithelial differentiation at this stage of development.

## Discussion

The PPB hypothesis posits a non-cell-autonomous mechanism for initial mesenchymal hyperplasia and predisposition to cancer initiation. In support of this, immunostaining showed segmental loss of epithelial DICER1 in Type I-II PPB cysts [18] suggesting that reduced DICER1 in lung epithelium contributes to the expansion of airspaces and the persistent proliferation of subepithelial mesenchyme. However, no downstream targets of DICER1 that could mediate these morphologic events were identified. We hypothesized that a plausible mediator of these morphologic events would be an epithelial gene that is expressed early in embryonic development, is suppressed by DICER1-cleaved mature miRNA(s) in late embryonic development, and has both cell autonomous and non-cell autonomous activities. FGF9 was considered a potential candidate because of its ability to promote mesenchymal proliferation and suppresses differentiation, and regulate epithelial branching [1–3, 20–22].

Analysis of Type I PPB tissue revealed prominent FGF9 expression in PPB-associated lung epithelium. In support of a causative role for FGF9 in mediating the pathogenic progression of PPB, we generated mice that congenitally lacked epithelial *Dicer1*. These mice showed increased *Fgf9* expression in lung epithelium and the development of histological and molecular phenotypes that mimicked Type I PPB. We also showed that inactivation of one or both alleles of *Fgf9* in Dicer1-deficient lung epithelium partially rescued the phenotype. Furthermore, the identification of specific miRNAs (miR-140 and miR-328) that regulate lung development, that show increased expression in lung epithelium as development progresses through saccular and alveolar stages, and that functionally suppress the *Fgf9* 3’ UTR, link Dicer1 activity to *Fgf9* mRNA regulation. These studies thus identify FGF9 as a biologically active downstream target of DICER1 that can serve as an initiating factor for PPB pathogenesis in humans that have germline and somatic mutations causing focal loss of lung epithelial *DICER1*.

### miRNA regulation of *FGF9*

We have shown that several conserved miRNAs can regulate the human and mouse *FGF9* 3’ UTR and that miR-140 regulates *Fgf9* expression in developing lung. However, the relationship between miRNAs and *Fgf9* may also have a role in the development and pathogenesis of other tissues. Mice that lack miR-140 are viable but exhibit decreased growth of long bones, attributed to reduced chondrocyte proliferation [35]. miR-140 is contained within intron 16 of the ubiquitin ligase, Wwp2, which is expressed in chondrocytes and in epithelial tissues, including lung [36, 37]. Although *Fgf9* was not identified as a target of miR-140 in chondrocytes, FGF9 is known to functionally regulate bone growth in part by suppressing chondrocyte proliferation [38] and could therefore be a functional miR-140 target in developing bone. Interestingly, miR-140 and *Wwp2* are both directly induced by Sox9 in chondrocytes, ATDC5 cells, and 293T cells [37]. Sox9 expression in distal lung epithelium [39] and the established role for Sox9 in chondrogenesis, suggests potentially interesting parallels between skeletal and lung development.

miR-140 is also involved in the pathogenesis of several human malignancies, including breast, ovarian, non-small cell lung, basal cell, colon, osteosarcoma, and hepatocellular carcinoma [40–46]. In hepatocellular carcinoma, miR-140 functions as a tumor suppressor, where it directly suppresses *Fgf9* expression [42]. In non-small cell lung carcinoma, miR-140 suppresses tumor growth and metastasis by downregulating IGF1R [41], and in breast cancer, miR-140 targets Sox2 [44]. Interestingly, *Fgf9* is expressed in 10% of human non-small cell lung carcinomas and induced expression of *Fgf9* in adult mouse lung epithelium leads to the rapid formation of adenocarcinomas [47, 48]. Thus, miR-140 suppression of *Fgf9* may not only be important for the development of lung and other tissues, but it may also function as an important tumor suppressor to ensure the quiescence of *Fgf9* in adult tissues.

Increasing evidence suggests that miR-328 also functions as a tumor suppressor in several types of cancers, including malignant glioma, breast, and colorectal carcinomas [49–52]. In malignant gliomas (World Health Organization grade IV astrocytic glioblastomas), miR-328 expression is decreased and is associated with worse prognosis [50]. Additionally, miR-328 showed reduced expression when comparing levels in grades II and III astrocytoma to those in secondary grade IV glioblastomas [49]. *Fgf9* is a potent growth factor for glial cells and was originally isolated from a glioma cell line [53]. Insufficient miR-328 in glioblastomas could lead to increased FGF9 expression and thus provide a mechanism to promote disease progression.

The human and mouse *FGF9* 3’ UTR are highly conserved and are similarly regulated by miR-140, miR-182, miR-183, miR-328. However, the human *FGF9* 3’ UTR differs from the mouse UTR in that it contains a microsatellite sequence and binding site for the RNA binding proteins FUBP3 and HuR. FUBP3 has been shown to potentiate *FGF9* mRNA levels [54]. Although the mouse *Fgf9* 3’ UTR does not contain an HuR binding motif, FGF9 was shown to regulate HuR expression and HuR was shown to regulate lung branching morphogenesis through regulation of *Fgf10* and *Tbx4* expression [55]. Thus, these RNA binding proteins, miRNAs, and the *FGF9* gene (including its protein product and 3’ UTR) may be involved in a common gene regulatory network that controls human lung development.

### Mechanisms of sarcomatous progression in PPB

Sarcomatous progression of the mesenchymal cells in Type I or cystic PPB appears to require bi-allelic mutations in *DICER1*. These mesenchymal cells typically have one allelic loss of function mutation and one somatic RNase IIIb missense mutation, leading to an inability to process mature 5p miRNAs, but preservation of 3p miRNAs [56]. Additionally, evidence was found for TP53 inactivation occurring as a third genetic event in PPB in the solid sarcomatous foci of the Type II and Type III neoplasms [56, 57]. The mesenchymal hyperplasia and either the increased proliferative index or increased number of mesenchymal cells resulting from *Fgf9* activation in PPB-associated epithelium, coupled with second hit *DICER1* RNase IIIb point mutations, could further enhance the oncogenic transformation of these mesenchymal cells.

### Effectiveness of mouse models for PPB

Inactivation of *Dicer1* in developing mouse lung using the *Shh-Cre/+* driver effectively models the earliest stages of PPB. Similarities include increased *Fgf9* expression, mesenchymal hyperplasia, and cystic expansion of epithelial ducts. However, this mouse model does not recapitulate the disease progression seen in some examples of human PPB. This is likely due to the severity of the phenotype of the mouse model after E14.5 and the non-viability of these mice after birth. To examine the effects of FGF9 expression at later stages of development that better match the more advanced stages of human PPB, we used an inducible *Fgf9* transgenic system. Activation of *Fgf9* from E16.5 to E18.5 showed marked similarities to human PPB at both the histological and molecular levels. An additional difference between the *Shh-Cre/+*, *Dicer1* ^*f/f*^ mouse model and PPB is that in familial PPB, *DICER1* is haploinsufficient in all cells and lost in lesion-associated epithelium, whereas in the mouse model, *Dicer1* is only inactivated in lung epithelium. Future refinements of the mouse model will be needed to reflect these differences in *Dicer1* genetics, the multifocal nature of human PPB, and the ability to observe disease progression beyond initial disease stages.

### Therapeutic potential

Manipulation of miRNA expression as a therapeutic target is under consideration for a wide range of human cancers [58]. Early stage PPB may be a particularly good target for miRNA directed therapy because sarcomatous progression in PPB, when it occurs, typically does so in the first five years of life [16]. Thus, inhibition of key targets of miRNAs during early childhood could slow progression or prevent events in the development of the cystic stage of PPB until after this developmental window of susceptibility. In the case of *Fgf9*, it appears that miRNAs serve to downregulate *Fgf9* expression during the transition from pseudoglandular to canalicular stages of development. In adult lung tissue, *Fgf9* expression is very low and may be maintained in this low stage independent of miRNA regulation. Consistent with this model, a recent study showed that loss of lung epithelial Dicer1 at later stages of development does not result in PPB-like cystic morphology [59]. Furthermore, ectopic activation of *Fgf9* in adult lung results in the rapid formation of adenocarcinoma, without associated mesenchymal hyperplasia [47]. This suggests that adult mouse lung mesenchyme becomes non-responsive to FGF9. Our demonstration that miR-140 and miR-328 mimics can directly suppress the *Fgf9* 3’ UTR, shows the therapeutic potential of supplying critical microRNAs directly to lung epithelium during the period of childhood susceptibility to PPB.

## Materials and Methods

### Animals

All mouse strains, including *Fgf9* ^*f/f*^, *Dicer1* ^*f/f*^, *Shh* ^*Cre/+*^, *Tre-Fgf9-Ires-eGfp*, *Sftpc-rtTA*, (*f*, floxed allele), have been previously described [2, 23, 24, 60, 61]. For conditional inactivation of Dicer1 and *Fgf9* in lung epithelium, mice were generated with the genotype, *Shh* ^*Cre/+*^, *Dicer1* ^*f/f*^, *Fgf9* ^*+/+*^ and *Shh* ^*Cre/+*^, *Dicer1* ^*f/f*^, *Fgf9* ^*f/+*^. Control mice were of the genotype *Shh* ^*Cre/+*^ *; Shh* ^*Cre/+*^, *Fgf9* ^*f/+*^; *Shh* ^*Cre/+*^, *Dicer1* ^*f/+*,^ *Fgf9* ^*f/+*^; *Dicer1* ^*f/+*,^ *Fgf9* ^*f/+*^ *;* or *Fgf9* ^*f/+*^, all of which showed no phenotypic differences from wild type mice. All loss of function mice were maintained on a mixed 129SV/J-C57BL6/J background. Transgenic strains, used for gain-of-function experiments, were maintained on the FVB background.

### Histology and immunohistochemistry analysis of human and mouse tissue

The human Type I Pleuropulmonary Blastoma tissue samples, formalin fixed and paraffin embedded, were obtained through the genetic studies tissue bank of the International PPB registry (http://www.ppbregistry.org/enrollment/genetic-studytissue-bank). Mouse embryo tissues were collected in ice cold PBS, fixed in 4% PFA overnight at 4°C, washed with 1X PBS, photographed, and embedded in paraffin prior to sectioning at 5 μm. For histology, mouse and human sample slides were stained with hematoxylin and eosin (H&E). For immunohistochemistry, paraffin section or cryo-sections were rehydrated and treated with 0.3% hydrogen peroxide in methanol for 15 min to suppress the endogenous peroxidase activity. Antigen retrieval was achieved by microwaving the sections in 10 mM citrate buffer for 10 min followed by gradual cooling to room temperature. Sections were incubated overnight at 4°C with the following primary antibodies: NKX2.1 (M3575, DAKO, 1:200); FGF9 (AF-273-NA, R&D, 1:100); Ki67 (VP-K451, VECTOR Laboratories, Inc., 1:200); p-ERK (4370s, Cell Signaling Technology, Inc, 1:200); Surfactant Protein C (Sftpc, AB3786, EMD Millipore Corporation, 1:1,000); CC10 (sc9722, Santa Cruz, 1:200); Aquaporin 5 (AQ5, AB92320, Abcam, 1:200); T1α (128370, SDHB, 1:200) and pHH3 (H9908, Sigma, 1:200). The anti-goat (BA9500, 1:200) and anti-syrian hamster biotin-conjugated (107065–142, 1:200) antibody were from VECTOR and Jackson ImmunoResearch Lab, Inc., respectively. All other antibodies were visualized using Broad Spectrum (AEC) Kit (95–9743, Zymed Laboratories Inc.) for mouse samples (staining with red color) and Broad Spectrum (DAB) Kit (95–9643, Zymed Laboratories Inc.) for the human samples (staining with brown color). All staining patterns are representative of at least three cases of human samples or three mouse embryos.

For quantification of Ki67 and p-ERK immunostaining, at least three individual tissue samples were included. For each tissue sample, three different slides were stained and analyzed, and for each slide, three 10X fields were counted for immunostained cells per 100 epithelial or mesenchymal cells. Statistical analysis was based on the three original tissue samples.

### *In situ* hybridization

*In situ* hybridization probes were from the following sources: *Fgf9* [62], *Lef1* [63], *Wnt2a* (A. McMahon, Harvard University, Cambridge, MA, USA), *Sftpc* [64]. Digoxigenin-labeled LNA miRNA detection probes were obtained from Exiqon Inc. (Scrambled-miR, #99004–01), has-miR140-5p (#21309–05), has-miR-328 (#38156–05). miRNA *in situ* hybridizations were performed according manufacturer instructions (http://www.exiqon.com/ls/Documents/Scientific/miRCURY-LNA-miRNA-ISH-Optimization-Kit-manual.pdf). cDNA-based probes were synthesized and labeled with a kit from Roche Applied Science. Whole mount *in situ* hybridization was performed as described [2, 3]. Following color reaction and methanol dehydration, tissues were photographed and then cryo-sectioned (5 μm), mounted on slides and rephotographed. *In situ* hybridizations of tissue sections were performed as previously described [65]. All staining patterns are representative of at least three cases of human samples or three mouse embryos.

### Lung explant cultures

Lung explant cultures were performed as described [2]. E10.5 embryonic lungs were dissected and cultured on Transwell filters (Costar, Corning) for 48 hours at 37°C, 5% CO_2_. For miR inhibition with locked nucleic acids (LNA), E10.5 lung explants were cultured with a total final concentration of 100 nM LNA in culture media. To quantify mesenchymal thickness, explants were photographed and mesenchymal thickness was measured using Canvas X software. Data shown is representative of at least three independent experiments. *p* values were calculated using the Student’s *t*-test and plotted as mean ± SD. For whole mount *in situ* hybridization, explants were cultured for 48 hr with LNAs, the lung explants were then lifted from the filters, fixed with 4% PFA over night at 4°C, and then processed for whole mount *in situ* hybridization.

### Quantitative PCR

Total RNA was purified from Lung explant cultures or HEK 293T cells using Trizol Reagent (#10296–010, Life Technologies Corporation, USA) or RNeasy Plus Micro Kit (#74034, Qiagen Inc. USA). cDNA was synthesized using the iScript^TM^Select cDNA synthesis Kit (#170–8841, BIO-RAD Laboratories, USA). mRNA expression was measured using TaqMan Fast Advanced Master Mix (#4444557, Life Technologies Corporation, USA) and TaqMan assay probes. miRNA were purified using the miRVana miRNA Isolation kit (AM1561, Life Technologies Corporation, USA) and the TaqMan miRNA Reverse Transcription kit (#4366596, Life Technologies Corporation, USA). mRNA expression was normalized to either *HPRT* or *GAPDH*. miRNA expression was measured using TaqMan assay probes (mmu-miR-140-5p, #001187; mmu-miR-182 5p, #002599; mmu-miR-183, 5p 002269; mmu-mir-328-3p, #000543). Expression was normalized to endogenous U6 NA (#001973, Life Technologies Corporation, USA). All assays were run on an ABI 7500 Fast Real-Time PCR System. Technical triplicates were run for each sample. Data was analyzed using the ΔΔCT method.

### *FGF9* 3′ UTR analysis

The human *FGF9* 3’ UTR in the pEZX-MT01vector was purchased from Genecopoeia (Rockville, MD USA). The mouse *Fgf9* 3’ UTR (nt 997–1538 from clone NM_013518) was excised from a T7 vector using SacI (blunted with Klenow) and NotI enzymes, and cloned in the psiCHECK-2 vector at the PmeI and NotI sites. The inserted 3’ UTR was confirmed by DNA sequencing. HEK293T cells where grown to 70% confluence in 12 well tissue culture plates and transfected with 50 ng plasmid DNA and 10nM microRNA mimics in Optimem medium (2 ml) following Lipofectamine 2000 instructions. After 6 hr, the media was replaced with fresh media (DMEM, 10% FBS). After 48 hr, cells were harvested and Luciferase activity was assessed on a Lumat LB 9507 luminometer (Berthold Technologies) using the Dual-Luciferase Reporter 1000 Assay System (E1980, Promega) according to the manufacturer instructions. Each condition was assayed in triplicate and all experiments where performed at least two times.

A mutant version of the *Fgf9* 3’ UTR, in which the miR-140 seed sequences was deleted, was generated using the QuikChange XL Site-Directed Mutagenesis kit (Agilent Technologies) using primers listed in S1 Table.

### MiRNA mimics and antagonists

miRIDIAN microRNA mimics (Dharmacon) were used to increase mature microRNA expression in HEK293 cells. Mimics were added to culture medium at a final concentration of 10 nM. MicroRNA mimics used are listed in S2 Table.

Tiny LNA antimiR oligonucleotides were custom designed to target the seed sequence of microRNAs. Tiny LNAs were synthesized with a phosphorothioate backbone (Exiqon, See S2 Table for sequences). Tiny LNAs were transfected in HEK293T cells as described previously [31, 66] at a final concentration of 10 nM for *in vitro* validation experiments. For expression in lung explant cultures, tiny LNAs were added directly to the culture media at a total final concentration of 100 nM.

### Statistics

The data are reported as the mean ± SD and changes with p values less than 0.05 were considered to be statistically significant. Data was analyzed using the unpaired Student’s t test. Numbers of mice used per group per experiment are stated in the figure legends.

### Study approval

This study was carried out in accordance with the recommendations in the Guide for the Care and Use of Laboratory Animals of the National Institutes of Health. The protocol was approved by the Washington University Division of Comparative Medicine Animal Studies Committee (Protocol Number 20130201). All efforts were made to minimize animal suffering. Human tissues were obtained from the International Pleuropulmonary Blastoma (PPB) Registry (http://www.ppbregistry.org/). Human tissues were obtained from the International PPB registry with IRB approval from Children′s Research Institute, Children′s National Medical Center Human Research Protection Office; IRB #4603, renewed with IRB electronic study #Pro00000315.

## Supporting Information

S1 Table Synthetic oligonucleotides used for mutagenesis and sequencing. (DOCX)

S2 Table Sequence of mature miRNAs and detection assays. (DOCX)

S1 Fig Anterior views of gross dissections of control (*Shh* ^*Cre/+*^, *Dicer* ^*f/+*^) and *Shh* ^*Cre/+*^, *Dicer1* ^*f/f*^ lungs at E14.5 and E16.5. Note the cystic dilation of the epithelial ducts and the increased mesenchymal thickness. Scale bar: E14.5, 200μm; E16.5, 500μm.
(PDF)

S2 Fig Anterior views of gross dissections of control (*Shh* ^*Cre/+*^, Dicer1^*f/+*^, Fgf9^*+/+*^); *Shh* ^*Cre/+*^, *Dicer1* ^*f/f*^, Fgf9^*+/+*^ and *Shh* ^*Cre/+*^, *Dicer1* ^*f/f*^, *Fgf9* ^*f/f*^ lungs at E14.5. Note that the *Shh* ^*Cre/+*^, *Dicer1* ^*f/f*^, *Fgf9* ^*f/f*^ lungs are smaller than *Shh* ^*Cre/+*^, *Dicer1* ^*f/f*^, *Fgf9* ^*+/+*^ lungs, with reduced cystic dilation of epithelial ducts. Scale bar: 500μm.
(PDF)

S3 Fig Target sequences and probe design for analysis of miR-140 and miR-328 activity. (A) Pairwise alignment of human and mouse *FGF9* 3' UTR near the miRNA 140-5p target site (underlined). (B) Pairwise alignment of human and mouse *FGF9* 3' UTR near the miRNA 328-3p target site (underlined). (C) miR-140-5p target site in the mouse *Fgf9* 3’ UTR. (D) miR-140-5p target site in the human *FGF9* 3’ UTR. (E) miR-328-3p target site in the mouse *Fgf9* 3’ UTR. (F) miR-328-3p target site in the human *FGF9* 3’ UTR.
(PDF)

S4 Fig Regulation of the human *FGF9* 3’ UTR by miRNAs. (A) Relative luciferase activity (compared to control cel-miR-67) of the human *FGF9* 3’ UTR is repressed by co-transfection with miRNA mimics, miR-140, miR-183 and miR-328. (B) Relative luciferase activity (compared to control cel-miR-67) of the human *FGF9* 3’ UTR is activated by co-transfection with miRNA mimics, miR-24 and miR-182. (C) Repression of the *FGF9* 3’ UTR by miR-140 (solid bars) was blocked by engineering mutations in which the seed sequences for miR-140 was deleted (open bars).
(PDF)

S5 Fig Expression of miR-328 in E18.5 lung epithelium. Histological sections from an E18.5 wild type mouse lung hybridized with a scrambled LNA *in situ* probe (left) or with an hsa-miR-328 LNA *in situ* probe (right).
(PDF)

S6 Fig LNA transduction into lung explants. (A-D) Whole mount E12.5 lung explant treated with unlabeled control LNA (A) or LNA-antimiR-140 (labeled with 6-FAM) (C). (B, D) Corresponding images showing 6-FAM fluorescence in the LNA-antimiR-140 treated explant. Scale bar: 200 μm.
(PDF)
