# Supplementary material for: Fibroblast Growth Factor 9 Regulation by MicroRNAs Controls Lung Development and Links DICER1 Loss to the Pathogenesis of Pleuropulmonary Blastoma
Source: PLoS Genet. 2015 May 15;11(5):e1005242. doi: 10.1371/journal.pgen.1005242 (PMC4433140; doi:10.1371/journal.pgen.1005242)
Supplement: S1 Table — (DOCX) [file pgen.1005242.s001.docx]

## S1 Table. Synthetic oligonucleotides used for mutagenesis and sequencing.

| Primer | Sequence 5’ to 3’ |
| --- | --- |
| Del 140 mfgf9 F | TCACTTGAGCCCTTAAAACATATAAATGCTTTCATGCGGTG |
| Del 140 mfgf9 R | CACCGCATGAAAGCATTTATATGTTTTAAGGGCTCAAGTGA |
| Del 328 mfgf9 F | TGTAATAAATCAAGCAAGAGGCAGCCCTCCAGGAG |
| Del 328 mfgf9 R | CTCCTGGAGGGCTGCCTCTTGCTTGATTTATTACA |
|  |  |
| Sequencing |  |
| Psicheck2 F | GCTCCAGATGAAATGGGTAAGT |
| Psicheck2 R | CTCCGAATGAGAGTGTTTCGTT |
| pHygEGFP F | TCCTGCTGGAGTTCGTGA |
| pHygEGFP R | CATCTCCCCCTGAACCTG |
