# Supplementary material for: Fibroblast Growth Factor 9 Regulation by MicroRNAs Controls Lung Development and Links DICER1 Loss to the Pathogenesis of Pleuropulmonary Blastoma
Source: PLoS Genet. 2015 May 15;11(5):e1005242. doi: 10.1371/journal.pgen.1005242 (PMC4433140; doi:10.1371/journal.pgen.1005242)
Supplement: S2 Table — (DOCX) [file pgen.1005242.s002.docx]

# S2 Table. Sequence of mature miRNAs and detection assays.

| **Micro RNA** | **Assay** | **Source** | **Product or Accession Number** | **Sequence (5’ – 3’)** |
| --- | --- | --- | --- | --- |
| mmu-miR-140- 5p | TaqMan qPCR | Life Technology | 001187 | CAGUGGUUUUACCCUAUGGUAG |
|  | miRIDIAN mimic | Dharmacon | MIMAT0000151 | CAGUGGUUUUACCCUAUGGUAG |
| hsa-miR-140-5p | LNA In situ probe | Exiqon | 21309-05 | CTACCATAGGGTAAAACCACT |
| Tiny LNA-140 | LNA antagomer | Exiqon | Custom | AAACCACT |
| mmu-miR-328-3p | TaqMan qPCR | Life Technology | 000543 | CUGGCCCUCUCUGCCCUUCCGU |
| hsa-miR-328 | miRIDIAN mimic | Dharmacon | MIMAT0000752 | CUGGCCCUCUCUGCCCUUCCGU |
|  | LNA In situ probe | Exiqon | 38156-05 | ACGGAAGGGCAGAGAGGGCCAG |
| Tiny LNA-328 | LNA antagomer | Exiqon | Custom | GAGGGCCA |
| mmu-miR-328- 5p | TaqMan qPCR | Life Technology | 464662_mat | GGGGGGCAGGAGGGGCUCAGGG |
| Control  SnoRNA202 | TaqMan qPCR | Life Technology | 4427975 | GCTGTACTGACTTGATGAAAGTACTTTTGAACCCTTTTCCATCTGATG |
| cel-miR-67 | miRIDIAN mimic | Dharmacon | MIMAT0000039 | UCACAACCUCCUAGAAAGAGUAGA |
| Scramble-miR | LNA In situ probe | Exiqon | 99004-01 | GTGTAACACGTCTATACGCCCA |
| Tiny LNA-con | LNA antagomer | Exiqon | Custom | A**G**ACCGCT^#^ |

^#^ Bold/underline is a missense mutation in the miR-140 tiny LNA sequence.

Extra stuff

|  | Mature miR sequence | UTR target sequence | anti-miR Tiny LNA sequence | RNA tm | DNA tm |
| --- | --- | --- | --- | --- | --- |
| Control | N.A | N.A | 5' A**G**ACC**G**CT 3' | 79°C | 53°C |
| miR 140 | 5' CAGUGGUUUuacccuaugguag 3' | 5' AACCACT 3' | 5' AAACCACT 3' | 84°C | 71°C |
| miR 328 | 5' CUGGCCCUCucugcccuuccgu 3' | 5' AGGGCCA 3' | 5' GAGGGCCA 3' | 70°C | 82°C |
|  |  |  |  |  |  |

microRNA Seed sequence in caps. Tiny LNA oligos where custom synthesized with a fully phosphothioate backbone and all the bases are LNA bases. Control Tiny LNA was designed as a miss match of tiny LNA anti-miR-140. Tiny LNA anti-miR 140 was 5' label with 6-FAM Fluorescein.

## Supplementary Table X

|  | Seed sequence | LNA sequence | RNA tm | DNA tm |
| --- | --- | --- | --- | --- |
| Control | N.E | 5' A**G**ACCGCT 3' | 79°C | 53°C |
| miR-140 | 5' TCACCAAA 3' | 5' AAACCACT 3' | 84°C | 71°C |
| miR-328 | 5' ACCGGGAG 3' | 5' GAGGGCCA 3' | 70°C | 82°C |
|  |  |  |  |  |

Control Tiny LNA was designed as a miss match of tiny LNA anti miR-140. LNA tiny oligos where synthesized with a fully phosphothioate backbone and all the bases are LNA bases.
