# Supplementary figures and images for: Fibroblast Growth Factor 9 Regulation by MicroRNAs Controls Lung Development and Links DICER1 Loss to the Pathogenesis of Pleuropulmonary Blastoma
Source: PLoS Genet. 2015 May 15;11(5):e1005242. doi: 10.1371/journal.pgen.1005242 (PMC4433140; doi:10.1371/journal.pgen.1005242)

S1\_Fig.

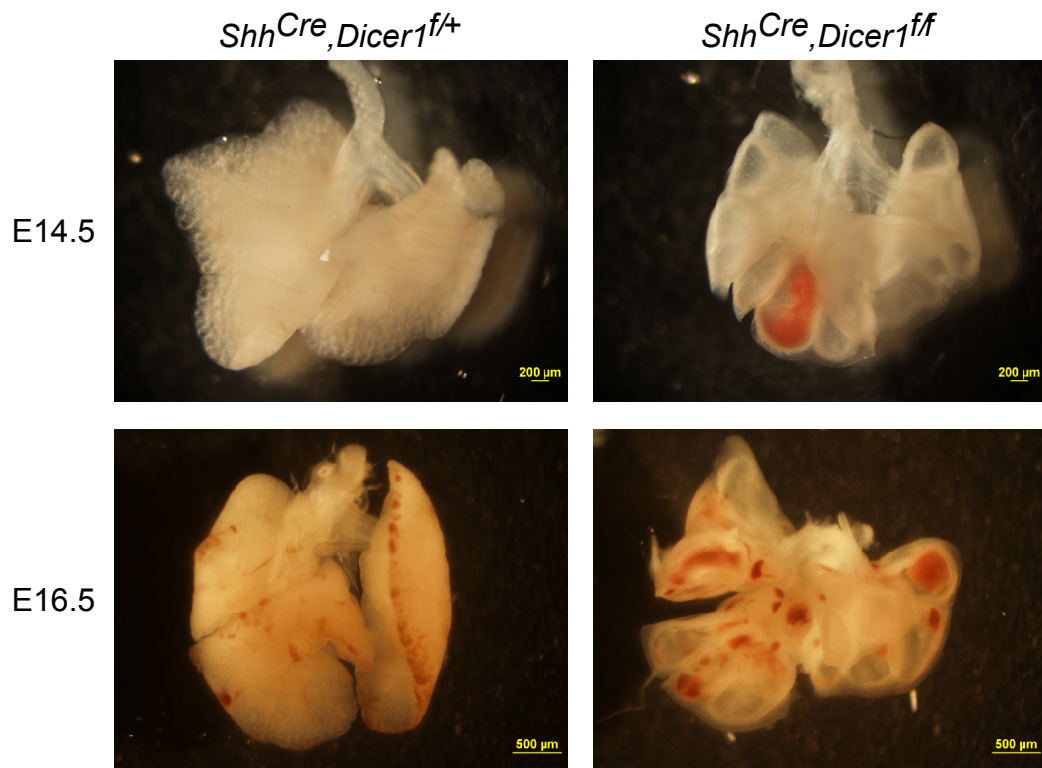

Supplement: S1 Fig — Note the cystic dilation of the epithelial ducts and the increased mesenchymal thickness. Scale bar: E14.5, 200μm; E16.5, 500μm. (PDF) [file pgen.1005242.s003.pdf]

S4\_Fig.

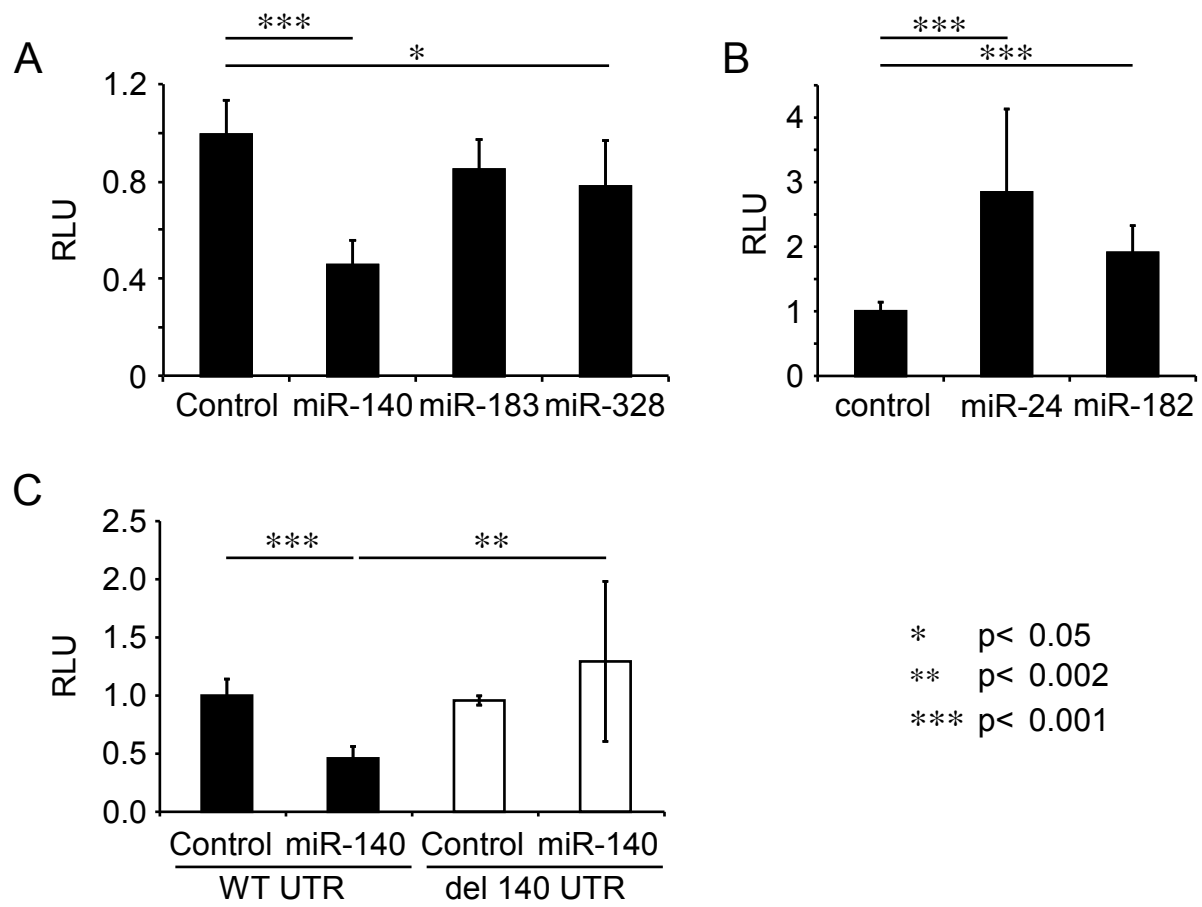

Supplement: S4 Fig — (A) Relative luciferase activity (compared to control cel-miR-67) of the human FGF9 3’ UTR is repressed by co-transfection with miRNA mimics, miR-140, miR-183 and miR-328. (B) Relative luciferase activity (compared to control cel-miR-67) of the human FGF9 3’ UTR is activated by co-transfection with miRNA mimics, miR-24 and miR-182. (C) Repression of the FGF9 3’ UTR by miR-140 (solid bars) was blocked by engineering mutations in which the seed sequences for miR-140 was deleted (open bars). (PDF) [file pgen.1005242.s006.pdf]

S5\_Fig.

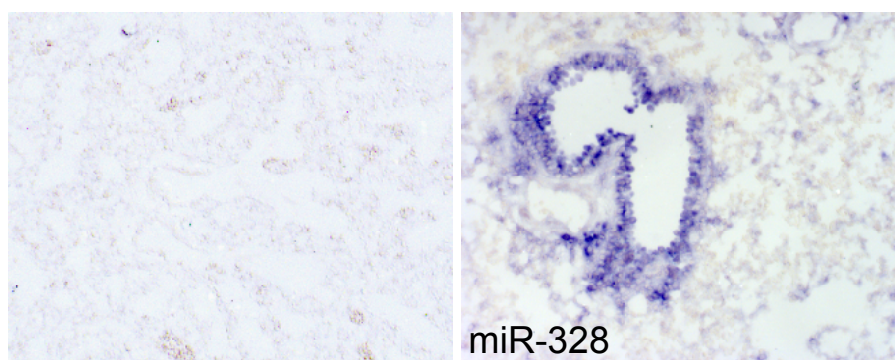

Supplement: S5 Fig — Histological sections from an E18.5 wild type mouse lung hybridized with a scrambled LNA in situ probe (left) or with an hsa-miR-328 LNA in situ probe (right). (PDF) [file pgen.1005242.s007.pdf]

S6\_Fig

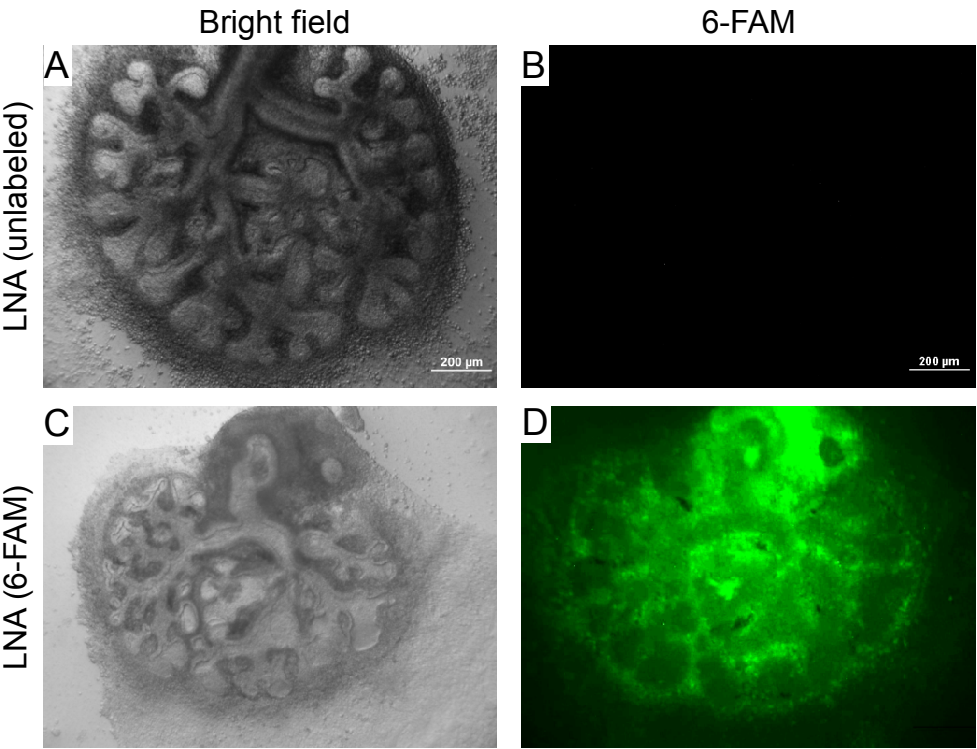

Supplement: S6 Fig — (A-D) Whole mount E12.5 lung explant treated with unlabeled control LNA (A) or LNA-antimiR-140 (labeled with 6-FAM) (C). (B, D) Corresponding images showing 6-FAM fluorescence in the LNA-antimiR-140 treated explant. Scale bar: 200 μm. (PDF) [file pgen.1005242.s008.pdf]
