# Supplementary material for: Fibroblast Growth Factor 9 Regulation by MicroRNAs Controls Lung Development and Links DICER1 Loss to the Pathogenesis of Pleuropulmonary Blastoma
Source: PLoS Genet. 2015 May 15;11(5):e1005242. doi: 10.1371/journal.pgen.1005242 (PMC4433140; doi:10.1371/journal.pgen.1005242)
Supplement: S2 Fig — Note that the Shh Cre/+, Dicer1 f/f, Fgf9 f/f lungs are smaller than Shh Cre/+, Dicer1 f/f, Fgf9 +/+ lungs, with reduced cystic dilation of epithelial ducts. Scale bar: 500μm. (PDF) [file pgen.1005242.s004.pdf]

S2_Fig.

Shh^{Cre},$Dicer1^{f/+}$ | Shh^{Cre},$Dicer1^{f/f}$ | Shh^{Cre},$Dicer1^{f/f}$,$Fgf9^{f/f}$

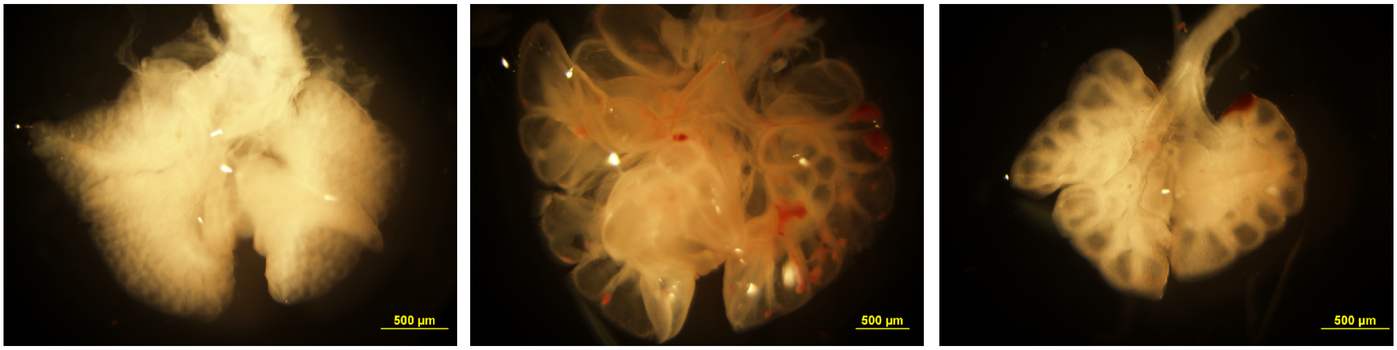

E14.5
