# Supplementary material for: Fibroblast Growth Factor 9 Regulation by MicroRNAs Controls Lung Development and Links DICER1 Loss to the Pathogenesis of Pleuropulmonary Blastoma
Source: PLoS Genet. 2015 May 15;11(5):e1005242. doi: 10.1371/journal.pgen.1005242 (PMC4433140; doi:10.1371/journal.pgen.1005242)
Supplement: S3 Fig — (A) Pairwise alignment of human and mouse FGF9 3' UTR near the miRNA 140-5p target site (underlined). (B) Pairwise alignment of human and mouse FGF9 3' UTR near the miRNA 328-3p target site (underlined). (C) miR-140-5p target site in the mouse Fgf9 3’ UTR. (D) miR-140-5p target site in the human FGF9 3’ UTR. (E) miR-328-3p target site in the mouse Fgf9 3’ UTR. (F) miR-328-3p target site in the human FGF9 3’ UTR. (PDF) [file pgen.1005242.s005.pdf]

S3_Fig.

A. **Pairwise alignment of human and mouse FGF9 3' UTR near the microRNA 140-5p target site.**

```
hFGF9 3'UTR 5' uugagcccuuaaaaaaguAACCACUauaaa-
gguuucacgcgguggguucuuauugauucgcugug
               ||||||||||||||.| |||||||||||||
|.|||||.||||||||||||||||||||.||.|||
mFgf9 3'UTR 5' uugagcccuuaaaaca-
uAACCACUauaaaugcuuucaugcgguggguucuuauugauuagcagug
```

B. **Pairwise alignment of human and mouse *FGF9* 3' UTR near the microRNA 328-3p target site.**

```
hFGF9 3' UTR  5'
cuccuggagggcugccuAGGGCCAcuugcuugauuuaucaugagagaagaggagagagagagaga

||||||||||||||||||||||||||||||||||||||.|..|.||.||.||||||||...|.|.
mFgf9 3' UTR  5'
cuccuggagggcugccuAGGGCCAcuugcuugauuuauuacaaaagcaggggagagaggcugcgg
```

C. **miR-140-5p target site in the mouse *Fgf9* 3' UTR**

```
5' cAGUGGUUUuacccuaugguag 3'      mmu-miR-140-5p

5' agcCCUUAAAACAUAACCACUa 3'      Fgf9
      ||  ||    | |||||||
3' gauGGUAUCCCAUUUUGGUGAc 5'      mmu-miR-140-5p
                |||||||
             5' AAACCACT  3'      anti-miR tiny LNA-140 sequence
```

D. **miR-140-5p target site in the human *FGF9* 3' UTR**

```
5' cAGUGGUUUuacccuaugguag  3'     hsa-miR-140-5p

5' agcCCUUAAAAAAGUAACCACUa 3'     FGF9
      ||  ||    |: |||||||
   gauGGUAUCCCAUU-UUGGUGAc 5'     hsa-miR-140-5p
                  ||||||||
              5' AAACCACT  3'     anti-miR tiny LNA-140 sequence
```

E. **miR-328-3p target site in the mouse *Fgf9* 3' UTR**

```
5' cUGGCCCUCucugcccuuccgu  3'     mmu-miR-328-3p

5' ccuGGAGGGCUGCCUAGGGCCAc 3'     Fgf9
      |:||||| |   |||||||
3' ugcCUUCCCGUC-UCUCCCGGUc 5'     mmu-miR-328-3p
                ||||||||
             5' GAGGGCCA   3'     anti-miR tiny LNA-328 sequence
```

F. **miR-328-3p target site in the human *FGF9* 3' UTR**

```
5' cUGGCCCUCucugcccuuccgu  3'     hsa-miR-328-3p

5' CCTCCUGGAGGGCUGCCUAGGGCCAc 3'   FGF9
         |:||||| |   |||||||
3'    ugcCUUCCCGUC-UCUCCCGGUc 5'   hsa-miR-328-3p
                   |||||||
                5' GAGGGCCA   3'   anti-miR tiny LNA-328 sequence
```

Yellow= microRNA binding site (MBS) or UTR target sequence
Cyan= microRNA Seed sequence
Green= anti-miR tiny LNA sequence
